# Unclassifiable non-CML classical myeloproliferative diseases with microcytosis: findings indicating diagnosis of polycythemia vera masked by iron deficiency 

**DOI:** 10.3906/sag-1907-67

**Published:** 2019-10-24

**Authors:** Elifcan ALADAĞ, Emine Arzu SAĞLAM, Salih AKSU, Haluk DEMIROĞLU, Nilgün SAYINALP, Hakan GÖKER, İbrahim Celalettin HAZNEDAROĞLU, Osman İlhami ÖZCEBE, Yahya BÜYÜKAŞIK1

**Affiliations:** 1 Department of Hematology, Faculty of Medicine, Hacettepe University, Ankara Turkey; 2 Department of Pathology, Faculty of Medicine, Hacettepe University, Ankara Turkey

**Keywords:** Polycythemia, microcytosis, myeloproliferative, iron deficiency

## Abstract

**Background/aim:**

Polycythemia Vera (PV) is a myeloproliferative disorder characterized by overproduction of morphologically normal red blood cells (RBCs), granulocytes, and platelets, a phenotype that is caused by a mutation (V617F) in Janus kinase 2 (JAK2). However, JAK2 V617F is also found in approximately 50% of patients with essential thrombocytosis and primary myelofibrosis, rendering its presence nonspecific as a diagnostic test. An increased red cell mass is a major criterion for the diagnosis of PV according to World Health Organization (WHO) 2016 criteria. High hemoglobin (Hgb) or Hematocrit (Hct) are universally used as indicators of an increased red cell mass for the diagnosis of PV. However, conditions such as iron deficiency (ID) with decreased mean cell volume may mask the diagnosis due to nonelevated Hct level. The aim of this study was to investigate the clinical characteristics of the patients with unclassifiable non-CML classical myeloproliferative disease with microcytosis (MPD/M) and nonelevated Hgb and Hct levels at diagnosis and to determine if some of these cases could be real PV cases masked due to ID-related microcytosis.

**Materials and methods:**

There were 23 MPD/M cases among 208 non-CML classical MPD cases (11%). Among 22 patients who had adequate test results related to the cause of microcytosis, ID and beta-thalassemia trait (TT) were the apparent causes of microcytosis in 15 and 1 cases, respectively.

**Results:**

Clinicopathological correlations revealed consistently positive JAK2 V617F mutation status (20/20, 100%), frequently elevated RBC count (17/23, 73.9%), and PV-compatible bone marrow findings (10/12, 83.3%). These findings are compatible with PV instead of essential thrombocytopenia or primary myelofibrosis. In spite of frequent cytoreductive treatment, 3 patients developed increased Hgb/Htc levels during median 58.2 (279–63) months’ follow-up.

**Conclusion:**

These data show that the majority of MPD/M cases are PV patients masked due to ID-related microcytosis.

## 1. Introduction

Myeloproliferative neoplasms (MPN) is a group of diseases associated with erythroid and myeloid cell proliferation leading to bone marrow hypercellularity, resulting in erythrocytosis, leukocytosis, and thrombocytosis in the peripheral blood [1]. The diagnosis of classical Philadelphia-negative myeloproliferative diseases (MPD) may represent a challenge for clinicians. Polycythemia vera (PV) is a myeloproliferative neoplasm with erythrocytosis predominance [2]. Radionuclide assessment is the historical gold standard method for the determination of total red blood cell mass (TRCM) [3]. Hematocrit (Htc) and/or hemoglobin (Hgb) values have long been recommended for the diagnosis of polycythemia vera (PV) since the World Health Organization (WHO) 2001 criteria mainly due to limitations of the radionuclide test [2,4]. High hemoglobin (Hgb) (>16 g/dL in females and 16.5 g/dL in males) or hematocrit (Hct) (>48% in females and >49% in males) are universally used as indicators of an increased red cell mass for the diagnosis of PV [2].  Hgb and Htc, as concentration values, may fail to reflect absolute erythrocytosis and increased TRCM in the case of hypervolemia/increased splenic sequestration (for instance, Budd Chiari syndrome (BCS)) and also in cases of concurrent microcytosis [5].

The aim of this study was to assess the clinical characteristics of the patients with MPD/M and not elevated Hgb and Htc levels at presentation. We hypothesized that many of those cases (to be mentioned as MPD/M from this point on) were true PV cases masked by microcytosis. 

## 2. Materials and methods

In our outpatient clinic at the Department of Hematology, Faculty of Medicine of Hacettepe University, ET, PMF, PV, and unclassifiable non-CML classical MPD patients diagnosed in 2003–2018 and with adequate electronical, clinical, and laboratory data were screened retrospectively. The MPD patients with deficient complete blood count test parameters at presentation were excluded. Patients could have been diagnosed with a MPD within 12 months before admission to our center. However, the patients who had received any cytotoxic/antiproliferative therapy were also excluded. The original diagnoses were presumably established depending on the universal diagnostic criteria valid at the time of presentation [6]. For this study elevated Hgb and Htc were accepted the same as WHO 2016 levels for the diagnosis of PV, i.e. 16 g/dL and 48% for women and 16.5 g/dL and 49% for men. An elevated red blood cell (RBC) count was accepted as >5.4 × 106/L and >6.0 × 106/L for females and males, respectively [2]. Microcytosis was defined as a mean cell volume lower than 80 fL. The patients with microcytosis (and not elevated Hgb/Htc values) at presentation were closely evaluated for all of the clinical and laboratory data also including the etiology of microcytosis, treatment modalities, disease course, and complications. Iron deficiency (ID) was accepted in case of a transferrin saturation (TS) <15% associated with a ferritin level <30 ng/mL. The ferritin cut-off level for iron deficiency was defined as 50 ng/mL in case of associated acute phase response and/or liver dysfunction. High-normal (>375 mcg/dL) or elevated (>450 mcg/dL) total iron binding capacity was also required for these patients with a ferritin level between 30 and 50 ng/mL [6,7]. Laboratory investigations were performed at Hacettepe University’s Clinical Biochemistry and Experimental Oncology Laboratories. Transferrin saturation was computed by dividing serum iron level by total iron binding capacity. For analysis the *JAK2* V617F mutation, genomic DNA extracted from the peripheral blood of the patients and DNA samples were tested with PCR and gel detection method (NuSieve 3:1 agarose/TBE gel) was used for amplification (InVivoScribe Technologies, San Diego, CA, USA). Bone marrow aspiration and biopsy specimens had been reported by experienced University staff hematopathologists and they were reevaluated by one of the authors considering current MPD marrow diagnostic criteria for the purpose of this study. Bone marrow findings were evaluated considering WHO diagnostic criteria for differential diagnosis of MPDs [8,9]. Bone marrow biopsy showing hypercellularity for age with trilineage growth (panmyelosis) including prominent erythroid, granulocytic, and megakaryocytic proliferation with pleomorphic, mature megakaryocytes (differences in size) was considered compatible with PV [8].

Data were presented as proportions or medians (minimum-maximum) for categorical and continuous variables, respectively. Categorical variables were compared using chi-square or Fisher’s exact tests and continuous variables were compared using Student’s t-test or Mann–Whitney U test. Statistical analyses were performed using SPSS (IBM Inc., Armonk, NY, USA) software versions 23.

This study was approved by Hacettepe University Ethical Board on 02.07.2019 with the approval number of GO 19672.

## 3. Results

There were 23 MPD with M cases among 208 non-CML classical MPD cases (11%) suitable for evaluation. Among 22 patients who had adequate test results related to the cause of microcytosis, ID and beta-thalassemia trait (TT) were the apparent causes of microcytosis in 15 and 1 cases, respectively. One patient had both iron deficiency and TT. Among 5 out of 22 patients without iron deficiency and/or TT 2 patients had clearly hypoferremia (a TS lower than 15%) and one patients had a borderline TS level (15.2%). Only 2 patients had clearly normal serum iron test results. Demographic and clinical characteristics are summarized in Table. All of 20 patients with available *JAK2* V617F mutation test result had *JAK2* V617F positivity. Nine of 23 MPD with M patients had an elevated red blood cell count at diagnosis. Serum erythropoietin level was available in 10 patients. It was decreased in 6 patients. Bone marrow biopsy was available in 18 MPD with M cases. After reevaluations bone marrow biopsy samples were found sufficient for MPD differential diagnosis in 12 patients. None of them were compatible with ET. PV and PMF were the possible diagnoses in 10 and 2 patients, respectively.

**Table:** Demographic and clinical characteristics of the MPD patients with microcytosis and normal Hgb/Hct levels at diagnosis.

	Mean(± standard deviation)
Age (years)	55.9 (±11.7)
Hemoglobin (g/dL)	12.4 (±1.9)
Hematocrit (%)	38.8 (±5.5)
Red blood cell (× 106/L)	5.4 (±0.86)
Platelet (× 103/L)	579.3(±377.4)
White blood cell (× 103/L)	11.8 (±6.1)
	(n) (%)
Female/male	15/8 (65.2/34.8)
Iron deficiency (yes/no/unknown)	16/6 (69.5/26)
JAK2 V617F mutation (yes/unknown)	20/3 (86.9/13.1)
Splenomegaly (yes/no)	14/9 (60.8/39.2)

Twenty-one patients received cytoreductive treatment (19 hydroxyurea, 1 pegylated interferon alpha-2a, and 1 thalidomide and ruxolitinib) after presentation. In spite of treatment, at a median follow-up of 31.5 (0–131.6) months, 3 out of 23 MPD with M patients developed elevated Hgb and/or Htc levels compatible with PV WHO 2016 diagnostic criteria either with or without iron supplementation. Eight out of 14 patients with normal RBC at diagnosis developed an increased red blood cell count. Only 6 patients did not show any lab sign indicating increased red blood cell production neither at diagnosis nor during disease course. Five of them had a suitable bone marrow biopsy specimen. The histology was compatible with PMF in 2 out of 5 patients.

Regarding important clinical complications; thromboembolism, hemorrhage, and transformation to myelodysplastic syndrome occurred in 9 (39,1%), 1 (4,3%), and 1 (4.3%), respectively, of the patients at a median follow-up duration of 2.4 months (0–15) after the presentation. All patients were still alive at the last contact.

## 4. Discussion

The results of our study demonstrated that MPD/M patients may actually have PV masked due to associated ID and microcytosis. Clinicopathological correlations revealed consistently positive *JAK2* V617F mutation status (20/20, 100%), frequently elevated RBC count (17/23, 73.9%), and PV-compatible bone marrow findings (10/12, 83.3%). These findings are compatible with PV instead of ET or PMF. Thrombohemorrhagic problems (9/23, 39.1%) are also more compatible with the diagnosis of PV [7]. In spite of frequent cytoreductive use 3 patients developed increased Hgb/Htc levels during median 58.2 (279–63) months’ follow-up.

It is clear that Hgb and Htc levels are suitable laboratory parameters reflecting TRCM in the absence of radionuclide TRCM testing [10]. However, these two routine blood parameters may be masked by two frequent complications of PV; hemodilution (as in BCS) and ID-related hypochromia/microcytosis. For such patients, WHO 2016 criteria proposed another alternative parameter for the determination of erythrocytosis: an increased red cell mass 25% above mean normal predicted value [1]. Although it may be useful in some cases, such as BCS patients with masked PV, this proposal is practically futile as this test is very rarely available. It is quite surprising that RBC count (which is not masked at least by ID-related hypochromia/microcytosis) had not previously been proposed as a diagnostic parameter in any established PV diagnostic criteria. Ideally the estimation power of RBC count in the differential diagnosis should be prospectively investigated in a larger cohort including various masked PV cases. Bone marrow biopsy may also be another guide in the differential diagnosis of the MPD with M cases, although there are contradictory study results regarding the reproducibility of bone marrow morphological features for the diagnosis of PV.

Currently, the MPD/M cases are usually followed as “unable to classify” in everyday clinical practice. Based on our present data, however, they should they be followed as true PV. This approach may have therapeutic impact in some cases as it has been confirmed that keeping Htc level below 45% with phlebotomy, hydroxyurea, or both decreases cardiovascular death and major thrombosis compared to a Htc target of 45% to 50% in PV [11]. 

In conclusion; in our study, the findings showed that the majority of MPD/M cases were PV patients masked due to ID related microcytosis. This clinical condition should be kept in mind in the diagnosis and treatment of patients with non-CML MPD.

## References

[ref0] (2016). The 2016 revision of WHO classification of myeloproliferative neoplasms: Clinical and molecular advances. Blood Reviews.

[ref1] (2018). Polycythemia vera: diagnosis, clinical course, and current management. Turkish Journal of Medical Science.

[ref2] (2004). Red cell mass measurement in polycythemia: Evaluation of performance and added value. Blood.

[ref3] (2007). Proposals and rationale for revision of the World Health Organization diagnostic criteria for polycythemia vera, essential thrombocythemia, and primary myelofibrosis: recommendations from an ad hoc international expert panel. Blood.

[ref4] (2016). Masked polycythaemia vera: presenting features, response to treatment and clinical outcomes. European Journal of Haematology.

[ref5] (2016). The 2016 revision to the World Health Organization classification of myeloid neoplasms and acute leukemia. Blood.

[ref6] (2014). In contemporary patients with polycythemia vera, rates of thrombosis and risk factors delineate a new clinical epidemiology. Blood.

[ref7] (2013). Evaluation of WHO criteria for diagnosis of polycythemia vera: a prospective analysis. Blood.

[ref8] (2016). The 2016 revision to the World Health Organization classification of myeloid neoplasms and acute leukemia. Blood.

[ref9] (2013). The diagnosis and management of erythrocytosis. BMJ.

[ref10] (2017). Risk of thrombosis according to need of phlebotomies in patients with polycythemia vera treated with hydroxyurea. Haematologica.

